# Germline deletion of β2 microglobulin or CD1d reduces anti-phospholipid antibody, but increases autoantibodies against non-phospholipid antigens in the NZB/W F1 model of lupus

**DOI:** 10.1186/ar4206

**Published:** 2013-03-27

**Authors:** Ram Raj Singh, Jun-Qi Yang, Peter J Kim, Ramesh C Halder

**Affiliations:** 1 Autoimmunity and Tolerance Laboratory, Division of Rheumatology, Department of Medicine, David Geffen School of Medicine at University of California Los Angeles (UCLA), 1000 Veteran Avenue, Los Angeles, CA 90095-1670, USA; 2 Department of Pathology and Laboratory Medicine, David Geffen School of Medicine at UCLA, 924 Westwood Blvd, Los Angeles, CA 90095, USA; 3 Jonsson Comprehensive Cancer Center, David Geffen School of Medicine at UCLA, Factor Building, Los Angeles, CA 90095, USA

## Abstract

**Introduction:**

β2-microglobulin (β2m) is required for the surface expression of MHC class I and class I-like proteins such as CD1d, Qa1 and neonatal Fc receptor (FcRn), all of which may impact the development of autoimmunity. Since CD1d is known to bind and present phospholipid antigens to T cells, we asked if the deficiency of β2m or CD1d will impact the development of anti-phospholipid antibodies as compared to other aspects of lupus autoimmunity.

**Methods:**

We introgressed the *β2m*-null genotype onto the NZB and NZW backgrounds for 12 to 14 generations to generate genetically lupus-susceptible (NZB/NZW)F1 (BWF1) mice that are β2m-deficient (β2m°). Circulating immunoglobulins (Ig), rheumatoid factor (RF), anti-DNA and anti-cardiolipin (anti-CL) antibodies, and renal disease were analyzed in these and CD1d-deficient (CD1d°) BWF1 mice that we had previously generated.

**Results:**

Whereas β2m° BWF1 mice had reduced serum IgG, they had increased mortality, nephritis, serum IgG anti-DNA antibody and RF as compared to heterozygous and wild-type littermates. These effects were recapitulated in CD1d° BWF1 mice, except that they also had increased serum IgG as compared to control littermates. Intriguingly, both β2m° and CD1d° mice had lower serum anti-CL antibody levels than in control littermates. Such CD1d dependence of anti-CL antibody production is not mediated by CD1d/glycolipid-reactive iNKT cells, as these cells reduced the production of RF and anti-DNA antibodies but had no effect on anti-CL antibodies.

**Conclusions:**

We report a novel dichotomous role of β2m and CD1d, whereby these molecules differently regulate autoimmunity against phospholipid versus non-phospholipid autoantigens.

## Introduction

Systemic lupus erythematosus (SLE) is an autoimmune disease characterized by uncontrolled production of autoantibodies against a variety of antigens such as nucleic acids and phospholipids, hypergammaglobulinemia and multi-organ inflammation [1]. Diverse sets of T-cells - CD4^+^, TCRαβ^+^CD4^-^CD8^-^, or γδ^+ ^T-cells - can promote autoantibody production [2-4]. The emergence of such autoreactive T helper cells in lupus is accompanied by impaired regulatory mechanisms, which include CD8^+^, invariant natural killer T (NKT) and γδ^+ ^T-cells [4-9]. Elucidating the role of antigen presenting molecules that present autoantigens to helper and regulatory T-cells would facilitate our understanding of the etiology and pathogenesis of lupus.

β2-microglobulin (β2m) is required for the expression of cell surface molecules, including classical major histocompatibility complex (MHC) class I, CD1, Qa-1, and FcRn (neonatal Fc receptor), and for the development of CD8^+^, NKT, and CD3^+^CD4^-^CD8^- ^T-cell subsets [10,11], all of which may potentially impact the development of humoral autoimmunity. In fact, several studies have used β2m-deficient (β2m°) mice to demonstrate a role of β2m-dependent events in the development of lupus. For example, β2m° NZB mice have reduced anti-erythrocyte antibodies and hemolytic anemia [12], and β2m° 129/J mice are resistant to an idiotype-induced experimental SLE [13]. β2m° MRL-lpr/lpr mice also exhibit decreases in anti-DNA antibody production, hypergammaglobulinemia and lupus nephritis [14-16]. These protective effects of β2m deficiency have been linked with the absence of FcRn [15], which is known to inhibit immunoglobulin G (IgG) catabolism [17,18]. However, lupus dermatitis is aggravated in β2m° MRL-lpr/lpr mice [16]. Mechanisms underlying such disparate effects of β2m-deficiency on autoimmune disease remain to be determined. Since β2m promotes the activation of CD8^+ ^and NKT cells via its association with MHC class I and CD1d, respectively, β2m deficiency may aggravate aspects of autoimmunity that are normally controlled by such potentially regulatory T-cells [5-7].

CD1d can also bind phospholipid antigens [19,20] and activate T-cells [21,22]. We reasoned that the absence of such CD1d-restricted self-phospholipid-reactive T-cells might result in the decreased production of anti-phospholipid antibody in β2m° and CD1d° mice. Here, we investigated the role of β2m on diverse aspects of lupus - survival, nephritis, hypergammaglobulinemia, rheumatoid factor (RF) and anti-DNA and anti-cardiolipin (anti-CL) autoantibodies - using a genetically susceptible animal model, namely NZB/NZW F1 (BWF1) mice that develop T-cell-dependent, autoantibody-mediated disease [23]. We show that β2m has distinct effects on diverse aspects of lupus autoimmunity.

## Material and methods

### Mice

The β2m° 129xC57BL/6 mice were crossed onto the NZB and NZW backgrounds (all from Jackson Laboratory, Bar Harbor, ME, USA) for 12 to 14 generations. At each backcross the heterozygous (β2m^**+/-**^) mice were identified by PCR using the neo [24] and β2m primers (sense, 5'-TATCAGTCTCAGTGGGGGTG-3'; antisense, 5'-CTGAGCTCTGTTTTCGTCTG-3'). The N12 β2m^**+/- **^NZB mice were crossed with N12 or N14 β2m^**+/- **^NZW mice to establish β2m^**+/+**^, β2m^**+/-**^, and β2m^**-/- **^(β2m°) BWF1 mice. The CD1d^**-/- **^(CD1d°) BWF1 mice were generated by crossing N10 CD1d^**+/- **^NZB mice with N12 CD1d^**+/- **^NZW mice [8]. The β2m° and CD1d° phenotypes were further confirmed by demonstrating absence of CD1d by flow cytometry of peripheral blood lymphocytes using an anti-CD1d monoclonal antibody, 1B1 (PharMingen, San Diego, CA, USA). To confirm that mice at the final backcross are indeed congenic, they were screened using a battery of simple sequence repeat markers (Jackson Laboratory), all of which discriminated congenic strains from the 129/B6 donors. Vα14^Tg ^BALB/c [25] and Jα18^-/- ^(Jα18°) BALB/c [26] mice were provided by Dr A Bendelac and Dr M Taniguchi, respectively. BALB/c SCID mice were purchased from Jackson Laboratory. All animal studies were performed according to the approved guidelines of UCLA Animal Research Committee.

### Assessment of lupus disease

Survival, renal disease, and autoantibody and IgG levels were assessed. Proteinuria was measured on a 0 to 4+ scale using a colorimetric assay strip (Albustix, Bayer, Elkhart, IN, USA). Severe proteinuria was defined as ≥300 mg/dl on two consecutive examinations [6]. Kidney sections were stained with H & E, periodic acid-Schiff (PAS), and Masson's trichrome, and scored in a blind fashion [27,28]. In brief, the mean scores for individual pathological features were summed to obtain the four main scores, including glomerular activity score (GAS), tubulointerstitial activity score (TIAS), chronic lesion score (CLS), and vascular lesion score (VLS) (Additional file 1, Table S1). These scores were converted into indices by dividing them by the number of individual features examined to obtain those scores. The indices thus obtained were then averaged and summed to determine a composite kidney biopsy index.

### Detection of autoantibodies

IgG anti-dsDNA antibody was measured by ELISA, as described [6], using serum samples diluted at 1:500 and the secondary antibody, alkaline phosphatase (AP)-conjugated goat-anti mouse IgG, at 1:1,000 dilution. Rheumatoid factor (RF, anti-IgG2a^a^) was determined by ELISA, as described [29], using serum samples diluted at 1:250 and the secondary antibody, AP-conjugated goat-anti mouse kappa at 1:1,000 dilution. Anti-CL antibodies were detected as previously described [30]. In brief, ELISA plates were coated with CL Ag (5 μg/ml, ≥97% TCL, Sigma, St. Louis, MO, USA) in 200-proof ethyl alcohol (Daigger, Vernon Hills, IL, USA). Vehicle (200-proof ethyl alcohol only) served as a control. Plates were then dried under a hood for 30 minutes and blocked with 1% BSA for 1 h at room temperature. Samples (serum dilution 1:200 to 1:500) and standard were added into plates for 2 h at room temperature. After washing, plates were incubated with AP-conjugated goat-anti mouse IgG (1:1,000) (Southern Biotechnology, Birmingham, AL, USA), developed with p-nitrophenyl phosphate substrate (Sigma, MO, USA) and optical density (OD) was determined at 405 nm using Multiskan (Thermo Labsystems, Pittsburgh, PA, USA). Normal BALB/c serum was used as a negative control and pooled serum from old BWF1 or MRL-lpr mice was used as a reference positive control. Total serum Ig and its isotypes were measured by a standard sandwich ELISA, using appropriate antibody pairs (Southern Biotechnology Associates, Birmingham, AL, USA), and serum samples diluted at 1:40,000 for total IgG and 1:20,000 for IgM and IgG isotypes. The secondary antibody, AP-conjugated goat-anti mouse IgG, IgM or IgG isotypes, was diluted at 1:1000.

### Reconstitution of SCID mice

BALB/c SCID mice were injected intraperitoneally (i.p.) with 5 μg LPS and 6 μg αGalCer separately and transferred intravenously (i.v.) with purified B cells (1 to 3 × 10^7^) isolated from 10-month-old Jα18° mice. These B-cell-reconstituted SCID mice were then transferred i.v. with enriched T-cells (3 to 6 × 10^6^) from donor 10-week-old Vα14^Tg ^or control Jα18° mice. Four days after the transfer, spleen cells harvested from these mice were analyzed for T-cell receptor β (TCR β) and αGalCer/CD1d-dimer^+ ^cells to verify the reconstitution of SCID mice with iNKT cells, as described previously [31]. As expected, the recipients of Jα18 T cells had no iNKT cells; and recipients of Vα14^Tg ^T cells had iNKT cells in their spleen. Spleen cells from these mice were cultured in complete medium without any further stimulation for 6 days. Culture supernatants were tested for IgG anti-DNA and anti-CL antibodies.

### Statistical analysis

Levels of antibodies and renal scores were compared using Student's *t*- or the Mann-Whitney *U*-test. Frequencies of antibodies and proteinuria were compared using two-sided Fisher's exact test. Survival was compared using a log-rank test.

## Results

### β2m deficiency in BWF1 mice accelerates lupus nephritis and reduces survival

**Figure 1:**
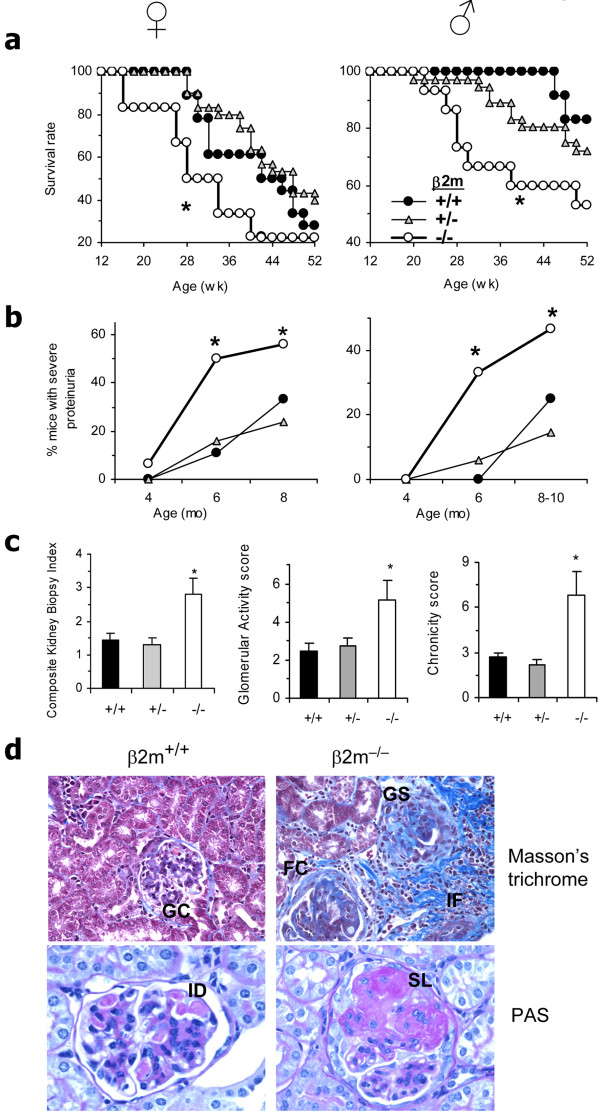
**β2 microglobulin (β2m) deficiency reduces survival and accelerates lupus nephritis in BWF1 mice**. We monitored 33 β2m^-/-^(β2m°) (18 female and 15 male, open circles), 66 β2m^+/- ^(30 female and 36 male, triangles) and 31 β2m^+/+ ^(18 female and 13 male, closed circles) littermates for the development of lupus. (**a**) Cumulative percent survival in β2m° BWF1 mice was compared with control β2m^+/- ^and β2m^+/+ ^littermate mice (*P <0.01 to 0.05, log rank test). (**b**) Cumulative percent mice with severe proteinuria (≥300 mg/dl protein on two consecutive occasions) was increased in β2m° mice compared to control littermates (*P = 0.02 to 0.05, Fisher's exact test). Differences were more pronounced (*P *<0.01) when β2m° mice were compared with all controls (β2m^+/- ^plus β2m^+/+^) mice. (**c**) Renal histological changes are expressed as the mean ± standard error of the composite kidney biopsy index and its components, glomerular activity and chronicity scores. Results from a representative experiment are shown (*P <0.05, β2m° vs control groups; *n *= 6 to 8 male 10-month-old mice per group, Student's *t*-test). (**d**) Representative kidney sections from these mice show severe kidney lesions with fibrosis (note increased aniline blue staining) in the glomeruli (glomerulosclerosis, GS), periglomerular region (fibrous crescent, FC) and interstitium (IF), interstitial inflammation, and large segmental lesions (SL) in β2m° mice, whereas the β2m^+ ^mice showed relatively less advanced lesions with increased glomerular cellularity (GC) and typical immune deposits (ID, see pink-colored deposits), but minimal or no GS or IF. PAS, periodic acid Schiff.

To investigate the role of β2m in the pathogenesis of diverse manifestations of lupus, we generated N12 β2m^**+/- **^NZB and N14 β2m^**+/- **^NZW mice and intercrossed them to generate the final β2m° BWF1 mice. As shown in Figure 1a, the cumulative survival was reduced in β2m° mice as compared with β2m^+/- ^and β2m^+/+ ^littermates (*n *= 31 to 66 animals per group). The reduction in survival in β2m° mice was associated with a higher frequency and earlier onset of severe proteinuria than in controls (Figure 1b). To further document the extent of renal disease exacerbation in β2m° mice, we scored stained renal sections (Figure 1c, d), which showed an increased composite kidney biopsy index as well as its components, glomerular activity and chronicity scores, in female (unpublished data) and in male mice that normally do not develop severe nephritis (Figure 1c). Vascular lesion scores, including thrombotic microangiopathy lesions, were not different among the three groups of mice (unpublished data). Thus, inflammation and fibrotic disease, but not vascular disease, were accelerated in β2m° mice.

### β2m deficiency in BWF1 mice reduces total serum IgG, but not IgM levels

**Figure 2:**
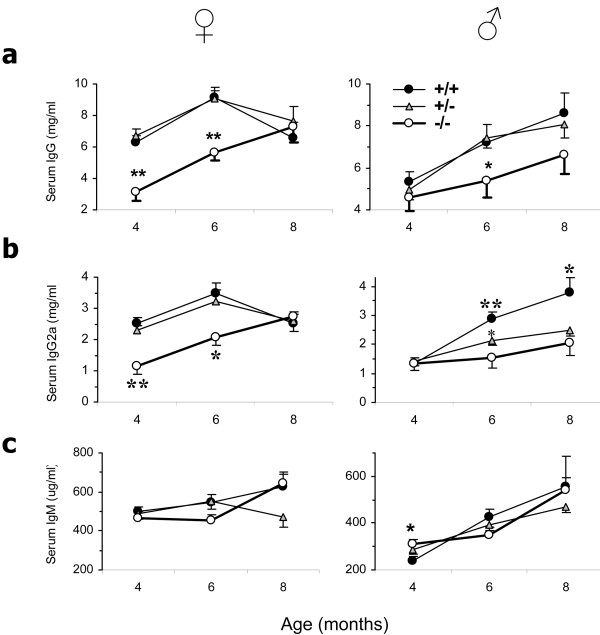
**Serum immunoglobulin (Ig) levels in β2 microgloblin-deficient (β2m°) BWF1 mice: reduced polyclonal total IgG, but not IgM**. β2m^+/+ ^(*n *= 18 to 22 female and 12 male, closed circles), β2m^+/- ^(*n *= 30 to 35 female and 28 to 35 male, triangles) and β2m° (*n *= 11 to 16 female and 10 to 16 male, open circles) mice were bled and sera tested for total IgG, IgG2a and IgM. Results are expressed as the mean ± standard error. (**a, b**) Serum levels of total IgG and IgG2a isotype were lower in β2m° mice than in control animals (*P <0.05, **P <0.01 to <0.001; Student's *t*-test). (**c**) Serum levels of total IgM were higher in β2m° than in β2m^+/+ ^mice (*P <0.05, Student's *t*-test).

Hypergammaglobulinemia is a feature of lupus. Thus, we expected to find increased IgG in β2m° BWF1 mice that experienced severe disease (Figure 1). However, β2m° BWF1 mice had reduced serum levels of total IgG and IgG2a as compared to β2m^+/- ^and β2m^+/+ ^littermates (Figure 2a, b). Serum levels of total IgM (Figure 2c), however, were unaffected in β2m° mice. Thus, β2m° BWF1 mice experience disease exacerbation at an age when they have low levels of total IgG and the IgG isotype of most pathogenic autoantibodies, IgG2a [32].

### β2m° BWF1 mice have enhanced anti-DNA antibody and RF levels

**Figure 3:**
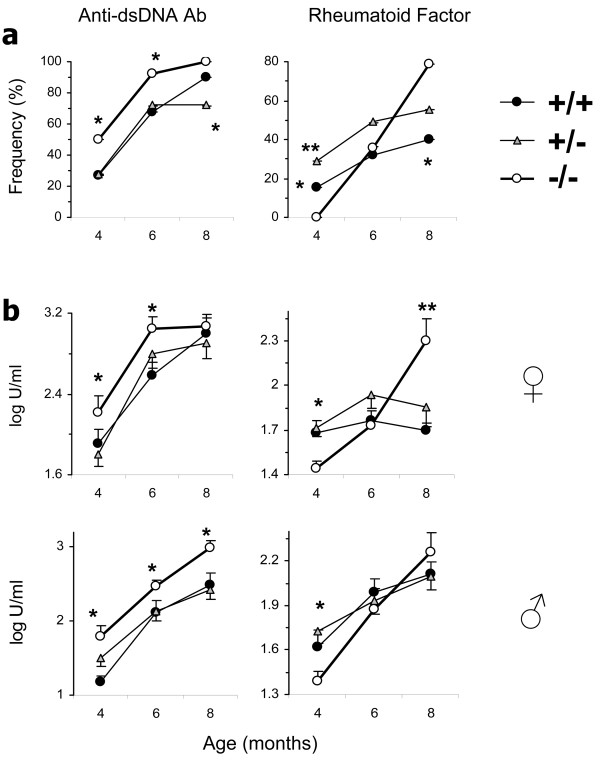
**Serum anti-dsDNA autoantibodies and rheumatoid factor (RF) are increased in β2 microglobulin-deficient (β2m°) BWF1 mice**. β2m^+/+ ^(*n *= 18 female and 12 male, closed circles), β2m^+/- ^(*n *= 30 female and 28 male, triangles), and β2m^-/- ^(*n *= 11 female and 10 male, open circles) mice were bled and serum samples tested for IgG anti-dsDNA antibody and RF. (**a**) Results are expressed as the percent of mice with autoantibodies, using a cutoff level of the mean + 4 SD optical density (OD) value in sera from six normal age-matched BALB/c mice. Results pooled from male and female mice are shown (*P <0.05, **P <0.01; Fisher's exact test). (**b**) Results of autoantibodies are shown as the mean ± standard error of log U/ml in male and female mice separately (*P <0.01 to 0.05, **P <0.001, Student's *t*-test).

Exacerbation of lupus, despite reduced IgG levels, in β2m° mice raised a possibility that they develop disease via a mechanism that is not dependent on IgG autoantibodies. However, the frequency of positivity and serum levels of IgG anti-dsDNA antibody were higher in β2m° mice than in control mice (Figure 3a, b). Male BWF1 mice, which normally do not develop autoantibodies in early life, had a marked increase in the prevalence of anti-dsDNA antibody (0% in β2m^+/+ ^vs 31% in β2m° mice at 4 months of age; *P *= 0.01; Fisher's exact test (data not shown)). Thus, anti-DNA B cells must be profoundly activated in β2m° mice from early life.

The frequency of positive RF and its levels in β2m° BWF1 mice showed a bimodal pattern, that is, its frequency and levels were lower than in β2m sufficient mice in early life, but the frequency and levels increased in β2m° mice to surpass the levels in the control littermates as the animals aged (Figure 3a, b). We surmise that the early decrease in RF in β2m° mice may be related to the absence of FcRn, whereas the increased RF in later life may be due to increased activation of RF-producing B cells.

### CD1d deficiency increases serum IgG and RF in BWF1 mice

**Figure 4:**
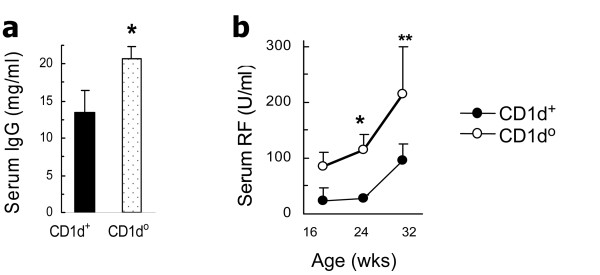
**Serum total immunoglobulin G (IgG) and rheumatoid factor (RF) are increased in CD1d-deficient (CD1d°) BWF1 mice**. CD1d° BWF1 mice and control littermates (CD1d^+/+ ^or CD1d^+/-^, designated as CD1d^+^) were bled and serum samples tested for total IgG and RF levels. **(a) **Eight-month-old CD1d^+ ^(*n *= 7) and CD1d° (*n *= 13) mice (*P = 0.04). (**b**) CD1d^+ ^(*n *= 8) and CD1d° (*n *= 15) mice (*P <0.01 and **P = 0.03 to 0.06). The negative control mean ± SD value in six normal BALB/c mice was 28.2 ± 8.6 U/ml for RF. Results are expressed as the mean ± standard error of the values.

The effects of β2m on lupus described above could be mediated by a variety of cell surface molecules, such as FcRn, MHC class I, Qa1 and CD1d, which require β2m for their optimal surface expression [10,11]. While reduced total IgG levels in the early life of β2m° mice (Figure 2) can be explained by the absence of FcRn, the disease exacerbation in β2m° BWF1 mice cannot be explained by FcRn-deficiency. Hence, we examined the effect of CD1d-deficiency on total IgG and autoantibody levels in the CD1d° BWF1 mice that we have generated [8]. We found that unlike β2m° BWF1 mice that had lower serum levels of IgG than control littermates (Figure 2), CD1d° BWF1 mice had significantly increased total serum IgG levels compared with CD1d^+ ^littermates (Figure 4a). Serum RF, which is not normally detected in high titers in BWF1 mice, was also increased in the CD1d° mice compared with CD1d^+ ^littermates (Figure 4b). Serum IgG anti-dsDNA antibody levels and lupus nephritis were also elevated in CD1d° BWF1 mice compared to controls (data not shown), as also reported previously [8]. Thus, the lack of a regulatory role of CD1d may explain, at least in part, the acceleration of lupus disease in β2m° BWF1 mice.

### Anti-CL antibody levels are reduced in β2m° BWF1 mice

**Figure 5:**
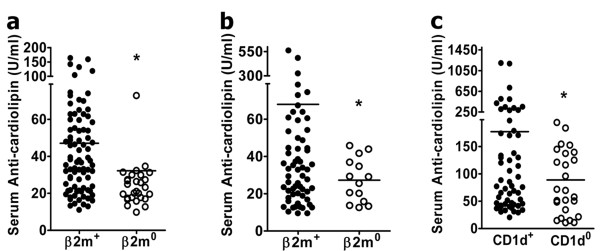
**Serum anti-cardiolipin (anti-CL) antibodies are decreased in β2 microglobulin-deficient (β2m°) and CD1d-deficient (CD1d°) BWF1 mice**. (**a-c**) β2m° or CD1d° BWF1 mice and control littermates (β2m^+/+ ^or β2m^+/-^, designated as β2m^+^; and CD1d^+/+ ^or CD1d^+/-^, designated as CD1d^+^) were bled and serum samples tested for serum immunoglobulin G (IgG) anti-CL antibodies: (**a**) 4-month-old β2m^+ ^(*n *= 88) and β2m° (*n *= 32); (**b**) 6- to 8-month-old β2m^+ ^(*n *= 75) and β2m° (*n *= 14); (**c**) 7- to 8-month-old CD1d^+ ^(*n *= 57) and CD1d° (*n *= 26) mice. Each symbol represents a single mouse, and horizontal bars represent the mean U/ml for the group. The negative control mean ± SD values for anti-CL antibody in six normal BALB/c mice was 15.1 ± 5.2 U/ml. *P <0.01, Student's *t*-test.

Preliminary analyses of autoantibodies using ELISA and western blot showed that a variety of antibodies against cellular and nuclear antigens were higher in β2m° BWF1 mice than in control littermates (data not shown). Surprisingly, however, no β2m° BWF1 mice had anti-CL antibodies above the cutoff level (mean + 4 (SD) OD in normal BALB/c mice). Subsequent analysis in a large cohort of mice showed that 6 to 10% of β2m° BWF1 mice compared to 36 to 39% of control littermates were positive for IgG anti-CL antibodies at different ages (*P *<0.01, Fisher's exact test). Levels of serum anti-phospholipid antibody were significantly lower in β2m° BWF1 mice than in control littermates (Figure 5a, b). These data suggest a contribution of β2m in the production of anti-CL antibodies in BWF1 mice.

### CD1d plays a role in the production of anti-CL antibody

CD1d can bind phospholipid antigens [19,20] and activate T-cells [21,22]. We reasoned that the absence of such CD1d-restricted self-phospholipid-reactive T-cells might result in the decreased production of anti-phospholipid antibody in β2m° and CD1d° BWF1 mice. Indeed, serum IgG anti-phospholipid antibody levels were reduced in CD1d° BWF1 mice compared with CD1d^+ ^littermates (Figure 5c).

**Figure 6:**
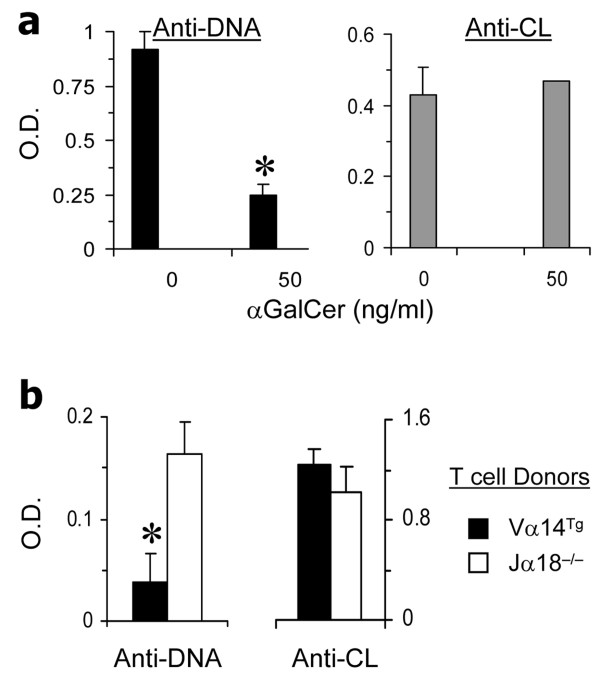
**Activated invariant natural killer T (iNKT) cells reduce anti-DNA antibodies, but not anti-cardiolipin (anti-CL) antibodies**. (**a**) *In vitro *studies. Spleen cells harvested from 5-month-old BWF1 mice were cultured with αGalCer for 5 days, and supernatants tested for immunoglobulin G (IgG) anti-DNA and anti-CL antibodies, shown as the mean ± SD optical density (OD); *P <0.05 to 0.01, *n *= 5. Results represent two similar experiments. **(b) ***In vivo *studies. BALB/c SCID mice (4-month-old) were reconstituted with purified B-cells from iNKT cell-deficient Jα18° BALB/c mice, and injected with lipopolysaccharide and αGalCer, as described in Methods. These mice were then implanted with T-cells from Vα14^Tg ^BALB/c mice or with T-cells from Jα18° BALB/c mice. Four days after the reconstitution, spleen cells were harvested and cultured without any further stimulation for 6 days. Culture supernatants were tested for IgG anti-DNA and anti-CL antibodies. Results represent two independent experiments, each using three mice per group. *P <0.05.

CD1d-restricted T cells comprise glycolipid-reactive iNKT cells that express the invariant TCR Vα14Jα18 and other NKT cells that do not express the invariant TCR. To determine the effect of iNKT cells on various autoantibodies, we cultured BWF1 spleen cells with glycolipid αGalCer. We found that while IgG anti-DNA antibody levels were reduced in the presence of αGalCer, IgG anti-CL antibody levels were unaffected (Figure 6a).

To further evaluate the differential effects of iNKT cells on anti-DNA versus anti-CL antibodies *in vivo*, we reconstituted BALB/c SCID mice with purified B cells from iNKT cell-deficient Jα18° BALB/c mice (to avoid any contamination with iNKT cells or iNKT cell-exposed B cells). These mice were then implanted with T-cells from Vα14^Tg ^BALB/c mice that have >50% T-cells as iNKT cells or with T-cells from Jα18° BALB/c mice that have no iNKT cells. As shown in Figure 6b, spleen cells from SCID mice implanted with iNKT cells produced lower levels of IgG anti-DNA antibody levels than spleen cells from SCID mice implanted with Jα18° T-cells. However, anti-CL antibody levels were unaffected by the presence or absence of iNKT cells. These data suggest that while glycolipid-reactive iNKT cells suppress anti-DNA antibody production, they do not affect the development of anti-CL antibodies.

## Discussion

Here, we show that BWF1 mice rendered deficient in β2m exhibit markedly increased renal disease with reduced survival, despite reduced serum IgG and IgG2a levels in early life. IgG anti-DNA antibody and RF are increased, but anti-phospholipid antibody levels are reduced in β2m° mice. All, but one, of these effects of β2m-deficiency may be explained, at least in part, by the absence of CD1d, with which β2m non-covalently associates, as CD1d° BWF1 mice also have accelerated nephritis, increased IgG anti-DNA antibody and RF, but reduced anti-phospholipid antibody levels. However, unlike β2m° mice, which have reduced serum IgG, CD1d° mice have increased serum IgG. Thus, β2m-deficiency may affect lupus via at least three possible mechanisms: 1) the effects of FcRn on IgG catabolism; 2) the immunoregulatory role of CD1d, and 3) the ability of CD1d to bind phospholipids to induce anti-phospholipid autoimmunity.

IgG antibodies comprise the major isotype responsible for humoral immunity and the pathological effectors of lupus [32]. The FcRn protects IgG from catabolism by diverting it from a degradative fate in lysosomes [17,18]. The IgG molecules of FcRn-deficient mice have an abnormally short half-life [17,18]. Because a functional FcRn molecule is dependent upon dimerization with β2m, β2m° mice also have reduced serum IgG [15,18]. Consistently, β2m° BWF1 mice have reduced serum IgG in pre- and early disease stages (Figure 2), but not in 8-month-old female (Figure 2) and male and female mice with terminal disease (data not shown). This lack of decrease in total serum IgG in older β2m° BWF1 mice could be due to a relative increase in IgG isotypes that bind weakly to FcRn and thus are less affected by the absence of FcRn. However, differences in the binding affinity of mouse FcRn for different mouse IgG isotypes are relatively small, with equilibrium dissociation constants (K_D_) of 0.42, 0.5 and 0.75 for IgG2a, IgG2b and IgG1, respectively [33]. Mammalian FcRn is specific for IgG and does not bind IgA, IgM and IgE. Consistently, serum IgM levels were unaffected in β2m° BWF1 mice. FcRn found on macrophages and dendritic cells can also facilitate the presentation of immune complexed antigens to T-cells [34,35]. Thus, the reduced antigen presentation and T-cell activation owing to FcRn-deficiency might contribute to the reduced IgG antibodies in β2m° mice.

The above effects of FcRn, however, do not explain lupus exacerbation in β2m° mice, which was severe enough to cause reduced survival (Figure 1a). Arguing against the role of FcRn in mediating the protective effect of β2m in lupus, the direct deficiency of FcRn did not affect survival in lupus-prone BXSB.*Yaa *mice [36]. Importantly, the effect of FcRn cannot explain an increase in anti-DNA antibodies in β2m° mice (Figure 3). Moreover, serum IgG increased as β2m° mice aged (Figure 2), despite the lack of FcRn that protects IgG against degradation. Serum levels of IgG2a that binds most avidly to mouse FcRn were also increased as the β2m° animals developed disease. Thus, a profound activation of autoreactive B-cells must occur in β2m° mice to have increased levels of circulating autoantibodies.

We have previously reported that tolerance in anti-dsDNA B-cells can be broken by autoreactive T-cells in non-autoimmune mice [5]. Such breakdown of tolerance is curtailed, however, by the emergence of T-cells that can inhibit autoantibody production [5]. These inhibitory T-cells are mostly CD8^+ ^T-cells that suppress autoantibody production via transforming growth factor β (TGFβ) [5,28] or B-cell ablation [6]. The latter, cytotoxic, CD8^+ ^T-cells recognize MHC class I-restricted peptides [6]. Expression of MHC class 1b molecule, Qa-1, by activated B-cells can also mediate CD8^+ ^T-cell suppression of immune responses [37,38]. In fact, the genetic disruption of the inhibitory interaction between CD8^+ ^T-cells and their target Qa-1^+ ^T-cells results in the development of autoantibodies and nephritis [39]. Thus, both classical and non-classical MHC class I molecules might contribute to disease protection in β2m-intact BWF1 mice.

In resonance with the above, the deficiency of MHC class I molecules H-2K and H-2D, of Tap1, which is required for the loading of processed peptides onto H-2K/D, or of CD8α, reduces survival in BXSB.*Yaa *mice [36]. However, the acceleration in mortality in BXSB.*Yaa *mice rendered deficient in H-2K/D, Tap1, or CD8α was not as profound as that observed in β2m° BXSB.*Yaa *mice [36], suggesting that more than one mechanism likely accounts for the protective effect of β2m in lupus.

Not all studies favor a protective role of MHC class Ia/b-restricted CD8^+ ^T-cells in lupus disease. For example, CD8-deficiency in NZB mice has been found to have no effect on anti-DNA antibody production [40]. The adoptive transfer of splenic CD8^+ ^T-cells into β2m° BWF1 mice also had no effect on disease in our preliminary study (data not shown). Thus, different mechanisms may account for the protective effect of β2m in different lupus-prone strains.

The disease-protective effects of β2m-dependent MHC class I proteins in BXSB.*Yaa *mice could be attributed to the additive functions of CD8^+ ^T-cells and IL-15 [36]. IL-15 also regulates the homeostasis and maturation of NKT-cells [41] that are restricted by CD1d, another β2m-associated molecule. Ample evidence suggests a regulatory role of CD1d-restricted T-cells in lupus and related diseases [8,24,31,42,43]. In fact, CD1d deficiency exacerbates nephritis and reduces survival in the hydrocarbon oil-induced and BWF1 models of lupus and dermatitis in MRL-*lpr *mice [8,24,42], although it has no effect on nephritis in MRL-*lpr *mice, or on survival in BXSB.*Yaa *mice [16,36,42]. CD1d-deficiency increases the production of many autoantibodies including anti-DNA, anti-OJ and anti-ribosomal P antibodies [8,24], and RF (Figure 4). Recent evidence also indicates a direct regulation of autoreactive B-cells by CD1d-reactive NKT cells [31,44]. Thus, it is reasonable to suggest that the protective effects of β2m against humoral autoimmunity and nephritis may be mediated, at least in part, via the regulatory effect of CD1d-reactive NKT-cells.

CD1d-reactive T-cells comprise heterogeneous populations of cells [45,46]. In a previous study, adoptive transfer of CD1d-reactive single positive T-cells induced a lupus-like disease in nude mice, whereas CD1d-reactive TCRαβ^+^CD4^-^CD8^- ^T-cells prevented the induction of autoimmunity [47]. Thus, some CD1d-reactive T-cells might protect against autoimmunity, whereas others might enhance autoimmune disease. Such diverse CD1d-reactive T-cells are likely to recognize different antigens and exert different functions.

Some CD1d-restricted T-cells can recognize phospholipid antigens bound to CD1d [21,22]. Function of these phospholipid-reactive T-cells is not understood. We demonstrated that serum anti-phospholipid antibody levels are reduced in β2m° and CD1d° mice (Figure 5). These data for the first time raise a possibility that CD1d presentation of self-phospholipids might induce anti-phospholipid autoantibodies, although further studies are needed to directly test this idea.

The reduction in anti-CL antibody levels in CD1d° mice was not due to a lack of anti-CL B-cell repertoire, as addition of lipopolysaccharide (LPS) to spleen cell cultures increased the levels of IgG anti-CL antibodies in these mice (Additional file 1, Figure S1). Thus, anti-CL B-cells exist in CD1d° mice, but they require CD1d for their activation *in vivo*.

We have recently reported that CD1d-restricted iNKT cells that respond to glycolipid αGalCer suppress the production of anti-DNA antibody and RF [31,48]. We asked whether such iNKT-cells promote anti-CL antibody production. In contrast to the effects of αGalCer on anti-DNA antibodies, anti-CL antibody levels were unaffected in BWF1 spleen cell cultures containing αGalCer (Figure 6a). Consistently, iNKT cells reduced IgG anti-DNA antibodies in SCID mice reconstituted with B-cells and iNKT-cells, but did not affect anti-CL antibody levels (Figure 6b). Thus, glycolipid-reactive type 1 iNKT-cells suppress the production of autoantibodies against non-phospholipid autoantigens, whereas non-iNKT cells, also called type 2 CD1d-restricted T-cells, might promote anti-CL antibody production.

Although this study used N10-N14 backcrossed mice that are expected to carry ≤0.1% genes from the 129/B6 β2m° or CD1d° founders, there remains the possibility that our results reflect the alteration of linked gene(s) during the backcross of the mutated β2m or CD1d 129 locus onto the lupus genetic backgrounds. Genotype analyses of our final backcrossed mice using simple sequence repeat markers, however, do not suggest a replacement with 129/B6 genes at any of the loci tested (data not shown). Moreover, differential regulation of different autoantibodies, increased anti-DNA and RF, and decreased anti-CL antibody, further suggests that the observed effects are not simply due to introgression of another gene that may have caused non-specific B-cell activation. Furthermore, similar data were obtained in more than one knockout strain, namely β2m° and CD1d° BWF1, arguing against the possibility that other lupus-susceptibility genes are responsible for our observations.

## Conclusions

**Figure 7:**
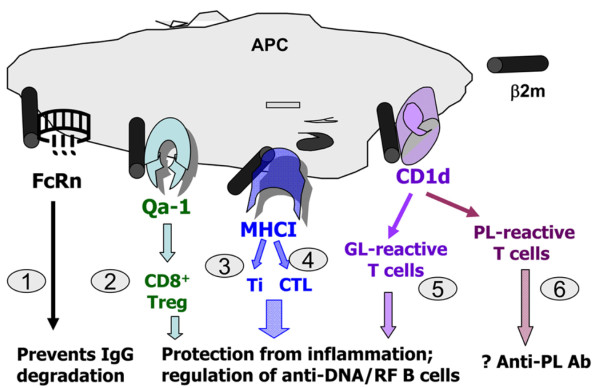
**Distinct roles of different β2 microglobulin (β2m)-associated glycoproteins in the regulation of humoral autoimmunity**. The findings in this report and previous studies suggest that β2m may affect lupus-like autoimmunity via at least six possible mechanisms: 1) neonatal Fc receptor (FcRn) effects on immunoglobulin G (IgG) catabolism [17,18] (Figure 2); 2) Qa-1-restricted CD8^+ ^regulatory or suppressor T-cells that can suppress autoimmunity [39]; 3) major histocompatability complex (MHC) class I-restricted CD8^+ ^Ti cells (inhibitory T-cells) that suppress autoantibody production via production of transforming growth factor β [5,28]; 4) MHC class I-restricted CD8^+ ^cytotoxic T lymphocytes (CTL) that can ablate autoreactive B-cells [6]; 5) protective role of CD1d-restricted glycolipid (GL)-reactive invariant natural killer T (iNKT)-cells in autoimmunity [8,24,31,42,44] (Figures 4 and 6a); and 6) the ability of CD1d to bind phospholipid (PL) antigens to induce anti-phospholipid autoimmunity (Figure 5). RF, rheumatoid factor; APC, antigen presenting cell.

Different MHC class I-related molecules associated with β2m play distinct roles in the development of different autoantibodies (Figure 7). A clear understanding of these roles may have implications for the development of novel therapies for the treatment of complex multi-system lupus disease. For example, inhibition or neutralization of FcRn may increase IgG catabolism [17], thus reducing the levels of pathogenic IgG autoantibodies, and the activation of regulatory CD8^+ ^or iNKT-cells may protect against autoimmunity. Patients with SLE and related diseases have reduced numbers and/or functions of CD1d-reactive T-cells [9,49-51], so the boosting of CD1d-reactive T-cells should be explored as a therapeutic strategy in SLE. In fact, treatment with rituximab restores the numbers and functions of CD1d-reactive T-cells to near-normal levels in patients with SLE [50]. There is a need for caution, however, as some CD1d-restricted T-cells might activate anti-phospholipid B cells and might induce or worsen anti-phospholipid syndrome, which manifests with vascular thrombosis and loss of pregnancy [52]. Further studies are needed to dissect the roles of CD1d-restricted glycolipid-reactive vs phospholipid-reactive T-cells in conferring the protective vs pathogenic roles in SLE.

## Abbreviations

αGalCer: α-galactosylceramide; anti-CL: anti-cardiolipin; β2m°: β2 microglobulin-deficient; BSA: bovine serum albumin; BWF1: NZB/NZW F1; CD1d°: CD1d-deficient; CLS: chronic lesions score; DN: double negative; ELISA: enzyme-linked immunosorbent assay; FcRn: neonatal Fc receptor; GAS: glomerular activity score; H & E: hematoxylin and eosin; IgG: immunoglobulin G; IL: interleukin; i.p.: intraperitoneal; i.v.: intravenous; LPS: lipopolysaccharide; MHC: major histocompatibility complex; NKT: natural killer T; OD: optical density; PAS: periodic acid-Schiff; PCR: polymerase chain reaction; RF: rheumatoid factor; SLE: systemic lupus erythematosus; TCR β: T-cell receptor β; TGFβ: transforming growth factor β; TIAS: tubulointerstitial activity score; VLS: vascular lesion score.

## Competing interests

The authors declare that they have no competing interests.

## Authors' contributions

RRS planned and supervised all aspects of this study. RRS, JY and RCH were involved in acquisition of data. All authors were involved in the analysis and interpretation of data. They all participated in the preparation of this manuscript and approved the final version of the manuscript.

## Supplementary Material

Additional file 1 **Table S1: A table showing the renal biopsy scoring system**. Scales for scores, indices and individual components. Figure S1: A figure showing that the reduction in anti-cardiolipin antibody levels in CD1d° mice is not due to a lack of anti-cardiolipin B cell repertoire in these mice. Anti-cardiolipin antibody levels in cultured supernatants of spleen cells stimulated with lipopolysaccharide (LPS) from wild-type (WT) and CD1d-deficient (CD1d°) mice. Click here for file
