# Supplementary material for: Germline deletion of β2 microglobulin or CD1d reduces anti-phospholipid antibody, but increases autoantibodies against non-phospholipid antigens in the NZB/W F1 model of lupus
Source: Arthritis Res Ther. 2013 Mar 27;15(2):R47. doi: 10.1186/ar4206 (PMC3672782; doi:10.1186/ar4206)
Supplement: Additional file 1 — Table S1: A table showing the renal biopsy scoring system. Scales for scores, indices and individual components. Figure S1: A figure showing that the reduction in anti-cardiolipin antibody levels in CD1d° mice is not due to a lack of anti-cardiolipin B cell repertoire in these mice. Anti-cardiolipin antibody levels in cultured supernatants of spleen cells stimulated with lipopolysaccharide (LPS) from wild-type (WT) and CD1d-deficient (CD1d°) mice. [file ar4206-S1.DOC]

###### Additional file 1

**Germline deletion of β2 microglobulin or CD1d reduces anti-phospholipid antibody, but increases autoantibodies against non-phospholipid antigens, in NZB/W F1 model of lupus**

Ram Raj Singh, Jun-Qi Yang, Peter J. Kim, Ramesh C. Halder

**Additional file 1, Table S1.** Renal biopsy scoring system

| Scores, indices and individual components | Scale |
| --- | --- |
| Glomerular activity score (GAS)  Glomerular hypercellularity  Karyorrhexis/fibrinoid necrosis  Cellular crescents  Inflammatory cell infiltrate  Hyaline deposits | 0-3+  (0-3+) × 2  (0-3+) × 2  0-3+  0-3+  Maximum = 21 |
| Tubulointerstitial activity score (TIAS)  Tubular cell pyknosis/nuclear activation  Tubular cell necrosis  Tubular cell flattening  Interstitial inflammation  Macrophages/epithelial cells in tubular lumen | 0-3+  0-3+  0-3+  0-3+  0-3+  Maximum = 15 |
| Chronic lesion score (CLS)  Mesangial matrix deposition  Glomerulosclerosis  Glomerular scars  Fibrous crescents  Tubular atrophy  Interstitial fibrosis | 0-3+  0-3+  0-3+  0-3+  0-3+  0-3+  Maximum = 18 |
| Vascular lesion score (VLS)  Arterial/arteriolar lesions | 0-3+  Maximum = 3 |
| Glomerular activity index (GAI) = GAS / 7  Tubulointerstitial index (TII) = TIAS / 5  Chronicity index (CI) = CLS / 6 |  |
| Composite Biopsy Index = GAI/7 + TII/5 + CI/6 | Maximum = 9 |

**Additional file 1, Figure S1.** **The reduction in anti-cardiolipin antibody levels in CD1d^o^ mice is not due to a lack of anti-cardiolipin B cell repertoire in these mice**. Spleen cells from CD1d^o^ BWF1 mice or control littermates (10-month-old) were cultured with or without LPS. Supernatants collected on day 5 were assayed for anti-cardiolipin antibodies. Results are representative of two independent experiments. This suggests that anti-cardiolipin B cells exist in CD1d^o^ mice, but they require CD1d for their activation in BWF1 mice.
